# Characterizing cognitive control abilities in children with 16p11.2 deletion using adaptive ‘video game' technology: a pilot study

**DOI:** 10.1038/tp.2016.178

**Published:** 2016-09-20

**Authors:** J A Anguera, A N Brandes-Aitken, C E Rolle, S N Skinner, S S Desai, J D Bower, W E Martucci, W K Chung, E H Sherr, E J Marco

**Affiliations:** 1 Department of Neurology, University of California, San Francisco, San Francisco, CA, USA; 2 Department of Psychiatry, University of California, San Francisco, San Francisco, CA, USA; 3 Akili Interactive Labs, Boston, MA, USA; 4 Department of Pediatrics, Columbia University Medical Center, New York, NY, USA; 5 Department of Pediatrics, University of California, San Francisco, San Francisco, CA, USA

## Abstract

Assessing cognitive abilities in children is challenging for two primary reasons: lack of testing engagement can lead to low testing sensitivity and inherent performance variability. Here we sought to explore whether an engaging, adaptive digital cognitive platform built to look and feel like a video game would reliably measure attention-based abilities in children with and without neurodevelopmental disabilities related to a known genetic condition, 16p11.2 deletion. We assessed 20 children with 16p11.2 deletion, a genetic variation implicated in attention deficit/hyperactivity disorder and autism, as well as 16 siblings without the deletion and 75 neurotypical age-matched children. Deletion carriers showed significantly slower response times and greater response variability when compared with all non-carriers; by comparison, traditional non-adaptive selective attention assessments were unable to discriminate group differences. This phenotypic characterization highlights the potential power of administering tools that integrate adaptive psychophysical mechanics into video-game-style mechanics to achieve robust, reliable measurements.

## Introduction

Cognition is typically associated with measures of intelligence (for example, intellectual quotient (IQ)^1^), and is a reflection of one's ability to perform higher-level processes by engaging specific mechanisms associated with learning, memory and reasoning. Such acts require the engagement of a specific subset of cognitive resources called cognitive control abilities,^2, 3, 4, 5^ which engage the underlying neural mechanisms associated with attention, working memory and goal-management faculties.^6^ These abilities are often assessed with validated pencil-and-paper approaches or, now more commonly with these same paradigms deployed on either desktop or laptop computers. These approaches are often less than ideal when assessing pediatric populations, as children have highly varied degree of testing engagement, leading to low test sensitivity.^7, 8, 9^ This is especially concerning when characterizing clinical populations, as increased performance variability in these groups often exceeds the range of testing sensitivity,^7, 8, 9^ limiting the ability to characterize cognitive deficits in certain populations. A proper assessment of cognitive control abilities in children is especially important, as these abilities allow children to interact with their complex environment in a goal-directed manner,^10^ are predictive of academic performance^11^ and are correlated with overall quality of life.^12^ For pediatric clinical populations, this characterization is especially critical as they are often assessed in an indirect fashion through intelligence quotients, parent report questionnaires^13^ and/or behavioral challenges,^14^ each of which fail to properly characterize these abilities in a direct manner.

One approach to make testing more robust and user-friendly is to present material in an optimally engaging manner, a strategy particularly beneficial when assessing children. The rise of digital health technologies facilitates the ability to administer these types of tests on tablet-based technologies (that is, iPad) in a game-like manner.^15^ For instance, Dundar and Akcayir^16^ assessed tablet-based reading compared with book reading in school-aged children, and discovered that students preferred tablet-based reading, reporting it to be more enjoyable. Another approach used to optimize the testing experience involves the integration of adaptive staircase algorithms, as the incorporation of such approaches lead to more reliable assessments that can be completed in a timely manner. This approach, rooted in psychophysical research,^17^ has been a powerful way to ensure that individuals perform at their ability level on a given task, mitigating the possibility of floor/ceiling effects. With respect to assessing individual abilities, the incorporation of adaptive mechanics acts as a normalizing agent for each individual in accordance with their underlying cognitive abilities,^18^ facilitating fair comparisons between groups (for example, neurotypical and study populations).

Adaptive mechanics in a consumer-style video game experience could potentially assist in the challenge of interrogating cognitive abilities in a pediatric patient population. This synergistic approach would seemingly raise one's level of engagement by making the testing experience more enjoyable and with greater sensitivity to individual differences, a key aspect typically missing in both clinical and research settings when testing these populations. Video game approaches have previously been utilized in clinical adult populations (for example, stroke,^19, 20^ schizophrenia^21^ and traumatic brain injury^22, 23, 24^); however, these are examples of using existing entertainment-based video games for assessment purposes rather than scientifically derived assessments that use video game mechanics for clinical assessments and/or training. This difference highlights the dissociation between two types of interactive digital media: those designed primarily for entertainment (‘video games') and those created for the purpose of cognitive assessment or enhancement.^6^ There are few examples of scientifically derived serious games used for clinical assessments, and, to the best of our knowledge, no examples of an entertainment-quality video game developed or validated for profiling cognitive abilities in clinical populations. Indeed, our previous work demonstrated the utility of incorporating adaptive algorithms in a video game for enhancing cognitive control,^18^ suggesting that similar cognitively targeted technology could be especially powerful in characterizing cognitive abilities in both healthy and clinical populations.

Here we administered a novel digital platform embedded with adaptive algorithms designed to assess cognitive control abilities associated with selective attention to children with and without a specific 16p11.2 BP4-BP5 deletion. This population was selected as children with the 16p11.2 BP4-BP5 deletion show high prevalence of inattention as well as language and social challenges when evaluated using clinical records and parent report tools.^25, 26^ Nineteen percent of deletion carriers meet criteria for attention deficit disorder,^27^ with 26% reaching DSM-IV-TR diagnostic criteria for autism spectrum disorders. Measurements of selective attention that involve distraction and inhibitory function like the Flanker task have shown attentional impairments in both attention deficit/hyperactivity disorder (ADHD)^28, 29, 30^ and autistic children.^31, 32^ However, the visual search task (which measures selective attention with distraction under an increasing distractibility load) has shown similar effects in children with ADHD,^33, 34, 35^ but the opposite effects in those with autism.^36, 37^ These finding epitomize recent meta-analysis findings by Karalunas *et al.*^38^ that suggest these types of assessments typically show small to moderate effect sizes when attempting to dissociate children with autism or ADHD to neurotypical controls. Despite evidence of clinically significant attention challenges in the 16p11.2 population, there has not yet been a study utilizing direct assessment measures of attention abilities in the presence of distraction.

Our study was predominantly conducted over the course of two 16p11.2 family meetings sponsored by the Simons Foundation in 2013 and 2015 with the exception of a single local family. The assessments were conducted in a semi-private conference room that allowed for a ‘real-world' testing environment but also inherently requires greater diagnostic sensitivity than testing in a controlled environment. These meetings facilitated our ability to recruit individuals with the 16p11.2 deletion who live throughout the world as well as their siblings who are age matched but do not carry the 16p11.2 deletion. Siblings represent an ideal comparison group due the sharing of 50% of their additional genetic material and the shared familial environment. We hypothesized that 16p11.2 deletion carriers would show slower and more variable reaction times, in accordance with historic literature from ADHD cohorts, compared with age-matched non-carrier siblings and unrelated healthy adolescents. We also hypothesized that this novel approach in assessing selective attention would reveal group-level deficits that traditional non-adaptive tests (Flanker and Visual Search) would not be able to uncover, given the modest sensitivity that non-adaptive platforms have shown in distinguishing between healthy and similarly affected populations.^38^

## Materials and methods

### Participants

One hundred eleven children participated in this study: 91 children (mean age 10.7 years±2.2, 41 females) who were not carriers for 16p11.2 deletion, and 20 children (mean age 10.1 years±3.0, 6 females) who were carriers for 16p11.2 deletion (Table 1). 16p.11.2 deletion carriers and their families were recruited from the broader Simons Variations in Individuals Project (VIP), where families across the world with this genetic disorder were invited to attend a family conference and participate in different research projects. In addition, 75 unrelated, unaffected children were recruited from ongoing work involving the characterization of attention abilities using a novel digital cognitive assessment platform (Project: EVO (EVO)), and tested in a traditional laboratory setting. This 75-person sample was included to provide a representative depiction of performance on this novel task in children (who are inherently variable from both an age and ability perspective), and determine whether any observed differences between affected and unaffected sibling could be replicated against a larger sample of unaffected children given the non-random convenient sample selection at the family meeting.

Forty-eight individuals of the total sample had IQ data with either the Differential Abilities Scales—2nd edition^40^ or the Wechsler Intelligence Scale for Children-IV^41^ through the broader Simons VIP (https://sfari.org/resources/autism-cohorts/simons-vip) and UCSF Sensory Neurodevelopment and Autism Program, with verbal IQ (VIQ) and non-verbal IQ (NVIQ) specifically assessed (Table 2). Legal guardians provided written informed consent, and minors gave assent to participate. The study was approved by the University of California, San Francisco Committee on Human Research. All subjects who took part at the family meetings were asked to complete two non-adaptive traditional assessments (Flanker and Visual Search) and one novel, adaptive assessment. Test administration occurred in semi-private rooms at the conference, with participants seated at tables with one administrator present to both explain and monitor each assessment. Headphones were provided to participants to decrease any environmental distractions and create a standardized testing experience for each participant. All assessments were administered using an iPad 2 in a counter-balanced manner.

### Project: EVO

**Figure 1:**
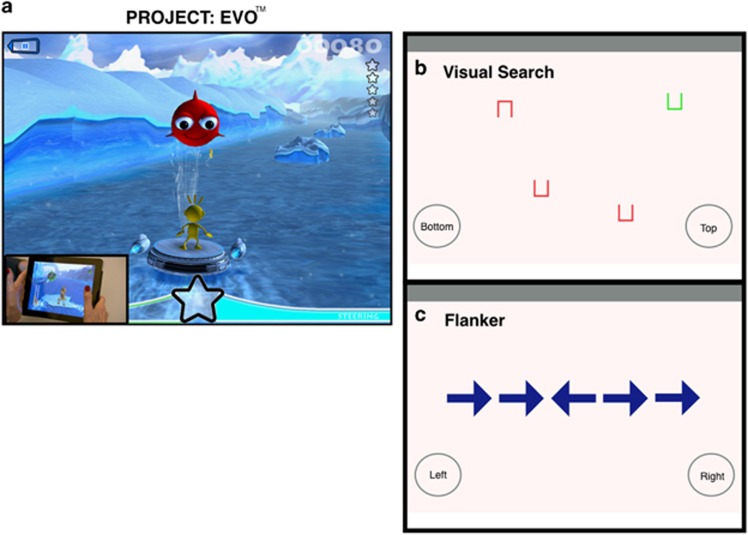
Screen shots of Project: EVO. (**a**) Image of participant playing Project: EVO showing the individual steering the character, while anticipating the appearance of target stimuli. (**b**) Image of the visual search task, (**c**) Image of the flanker task (incongruent trial type).

EVO is a digital cognitive assessment and intervention system developed by Akili Interactive Labs to assess and train cognitive control abilities in clinical populations through immersion in an adaptive, high-interference environment that is built to look and feel like a consumer-grade action video game (Figure 1a). EVO game development is based upon the principles utilized and validated by Anguera *et al.*^18^ in their development of a video game (NeuroRacer). EVO is designed for playing on iOS mobile devices using a consumer game engine (UNITY) with high-level art, music, feedback and storylines to ensure engagement with children and adolescents, whereas NeuroRacer was not developed with such assets or distribution protocols in mind. Similar to NeuroRacer, EVO assesses perceptual discrimination, while single- and multi-tasking (that is, while performing a simultaneous visuomotor tracking task). The perceptual discrimination task requires selective attention in the presence of distraction (with distraction here consisting of the visuomotor tracking component) to correctly respond to specific colored stimuli (tapping anywhere on the screen when a target appears), while ignoring all others, much like a standard ‘Go/NoGo' task. Visuomotor tracking involves navigating one's character through a dynamically moving environment with the goal of avoiding the walls and obstacles.

Unlike NeuroRacer, EVO utilizes adaptive algorithms to change game difficulty on a trial-by-trial basis (as opposed to the block-by-block approach used in NeuroRacer) for both the tracking task (adapting the speed of the forward path and sensitivity of the user's motions) and discrimination task (adapting the response window for a target), with real-time feedback making the participant aware of their performance. More specifically, the adaptive algorithm makes proportional changes in game play difficulty depending upon participants' performance from an 80% accuracy median, an approach comparable to that used in NeuroRacer. Althoguh most cognitive adaptive procedures include simple staircases, EVO employs rapidly adapting algorithms suited for measuring threshold accuracy in a fast-paced environment. The EVO assessments lasted a total of 7 min, during which time participants completed a specified number of both correct and incorrect trials (~100 trials, with an ITI of 1000 ms±500) allowing the adaptive algorithm to settle on a prescribed level of difficulty that would forecast discrimination accuracy. The outcome measures acquired from the EVO platform include: a calculated threshold level, mean response time and response time variability to perceptual targets during the single and multi-tasking conditions.

### Non-adaptive traditional cognitive assessments

We used a battery of validated neuropsychological cognitive tests integrated into an iOS tablet-format app to assess attention-based cognitive control in a comparable manner to that of EVO: (1) visual search task: participants were presented with an array of either 4 or 12 Landolt squares with an opening on one side until participants located the target (a green box with a gap in the top or bottom) and indicated the location of the gap (top, bottom) by tapping on a box with either ‘top' or ‘bottom' (Figure 1b). There are two distinct modes: a feature search where red Landolt squares were present in addition to the single green target; and a conjunction search where distractor boxes were green and red, with all green distractor boxes having gaps on either side, whereas red distractor boxes had gaps on the top and bottom similar to the green target box. Each task consisted of 100 trials, with inter-trial intervals jittered between 1200 and 1800 ms in 100 ms increments. Task performance was assessed by examining the mean response time (to correct responses) for each trial type possibility (set size: 4-item trials, 12-item trials; set type: feature search trials, conjunction search trials), with response time cost between target identification for feature and conjunction trials across each set size also determined ((cf. ref. 42); Visual Search cost=set size 12−set size 4 for feature and conjunction trials separately). (2) Flanker task: based on the original Eriksen and Eriksen task,^39^ participants responded to the direction of a central arrow, with flanking arrows either having the same (congruent) or different (incongruent) directionality (Figure 1c). The visual search and flanker tasks lasted a total of 7 min each, during which time the player watched an instructional video, played a practice round and completed the assessment. Task performance was assessed by examining response time to correct responses to each trial type, and calculating a cost between these trial types (cf. Lee *et al.*, 2012; Flanker Cost=Incongruent response time (RT)−Congruent RT). See Table 1 for a comparison of tasks.

### Statistical analyses

Analysis of covariances (ANCOVAs), covarying for age, were used to test for main effects and interactions between groups and measures, with planned follow-up *t*-tests (assessing equality of variance using Levene's Test for Equality of Variances) and the Greenhouse–Geisser correction used when appropriate. For the subsample of the participants with IQ measurements (see Table 2 for details by participant cohort), non-verbal IQ was also used as a covariate in a separate analysis (in addition to age) to test for main effects and interactions as above. All effect size values were calculated using Cohen's *d* (ref. 43) and corrected for small sample bias using the Hedges and Olkin approach.^44^

## Results

Twenty children with the same 16p11.2 BP4-BP5 deletion, 16 non-carrier siblings, and 75 unaffected unrelated children overlapping in age completed the EVO assessment. Eighteen deletion carrier and 19 non-carrier children (a mix of non-carrier siblings (14) and unrelated children (5)) completed the non-adaptive traditional assessments (the Flanker and Visual Search tasks). Comparison of IQ data from those 48 participants that were part of the larger Simons VIP project revealed both VIQ and NVIQ being significantly lower in deletion carriers than controls (Table 2). The mean carrier results for VIQ (84.3±11.4) and NVIQ (88.5±10.1) were in agreement with other Simons VIP Consortium study groups^27^ who have access to larger samples (*N*=85) and documented the average VIQ and NVIQ of deletion carriers to be 79±18 and 86.8±15.1, respectively.

### Diagnostic assessments: EVO Levels

#### Comparison of children with 16p11.2 deletion with their non-carrier siblings

Game play level for visuomotor tracking and perceptual discrimination during single-task and multi-tasking conditions reflects performance that approaches 80% accuracy. With respect to visuomotor tracking (for example, navigating without hitting walls or obstacles), a 2 (between factor: group) × 2 (within factor: condition) repeated measures ANCOVA examining the participant level for optimal tracking performance during single- and multi-tasking conditions revealed a group main effect (F(1,34)=17.19, *P*<0.001, Cohen's *d*=1.36) but neither an effect of condition (F(1,34)=1.26, *P*=0.27) nor an interaction (F(1,34)=0.74, *P*=0.40). This result suggests that children with 16p11.2 deletion played EVO at a lower visuomotor tracking level than their non-carrier siblings in each condition. Thus, multi-tasking did not differentially impair play for children with the 16p11.2 deletion. A similar analysis assessing the perceptual discrimination level (for example, responding to targets and ignoring distractors) during single- and multi-tasking conditions revealed a group main effect (F(1,34)=16.45, *P*<0.001, Cohen's *d*=1.03) but again no effect of condition (F(1,34)=0.19, *P*=0.66) or interaction (F(1,34)=0.91, *P*=0.35), suggesting carriers thresholded to a lower discrimination level than their non-carrier siblings. It should be noted that each of the primary effects of interest remain significant when Non-verbal IQ was used as an additional covariate in the ANCOVA analyses (F(1,18)⩾4.6, *P*⩽0.045), although this analyses should be considered exploratory given the sample size involved (Table 2).

#### Comparison of children with 16p11.2 deletion with unrelated neurotypical controls

Comparisons between children with 16p11.2 deletion and the unrelated neurotypical children also revealed a group main effect for both visuomotor tracking and perceptual discrimination level (F(1,92)⩾33.02, *P*⩽0.001, *d*⩾1.44 in each case), as well as a main effect of condition (single- vs multi-tasking; F(1,92)⩾10.10, *P*=0.002), but no condition by group interaction (F(1,92)⩽3.25, *P*⩾0.074 in each case). As above, the primary effects of interest remain significant when Non-verbal IQ was used as an additional covariate in the ANCOVA analyses (F(1,33)⩾9.5, *P*⩽0.004).

### Selective attention: EVO response time and response time variability

#### Comparison of children with 16p11.2 deletion with their unaffected siblings

**Figure 2:**
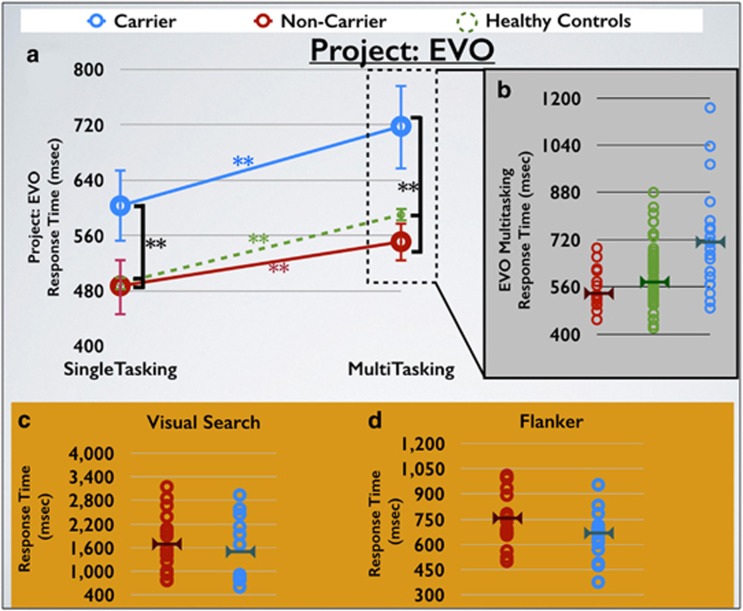
Project: EVO selective attention performance. (**a**) EVO single- and multi-tasking response time performance for each group (carriers, non-affected siblings and non-affected control groups). (**b**) EVO multi-tasking RT. (**c**) Visual search task performance for the conjunction 12 conditions (most difficult). (**d**) Flanker task performance for the incongruent trial type. Error bars represent s.e., horizontal bars on each plot represent the mean. **P<0.01. RT, response time.

In our next set of analyses, we assessed response time and response time variability on the perceptual discrimination task (that is, responding to specific targets and ignoring non-targets). These analyses echoed the level-based findings: response time to targets revealed a main effect of group (F(1,33)=15.50, *P*<0.001, *d*=1.29), but neither a main effect of condition (F(1,33)=0.12, *P*=0.74) nor a condition by group interaction (F(1,33)=0.98, *P*=0.33; Figures 2a and b). The same approach examining response time variability revealed a group main effect (F(1,33)=9.08, *P*=0.005, *d=*1.01), but neither a condition (F(1,33)=2.50, *P*=0.12) nor an interaction (F(1,33)=2.21, *P*=0.15), suggesting that children with the 16p11.2 deletion have greater performance variability than siblings regardless of condition. However, as expected, each group showed an increase in RT from the single-task to the multi-tasking condition (*P*<0.004 in each case). As a whole, these results suggest that the deletion carrier group showed slower response times that were magnified and more variable regardless of task complexity relative to their own siblings (There were no group differences with respect to false-positive rate for either condition (*P*>0.30 in each case)). The inclusion of Non-verbal IQ as an additional covariate in the ANCOVA analyses revealed a very modest trending effect for response time (F(1,20)=2.8, *P*=0.11), with a significant effect for response time variability (F(1,20)=5.5, *P*=0.029).

#### Comparison of children with 16p11.2 deletion with unrelated neurotypical controls

A similar pattern of effects was observed when comparing the deletion carrier children with the unrelated control group for both the response time and response time variability analysis. There was a main effect of group (F(1,92)⩾23.86, *P*⩽0.001, *d*⩾1.2 in each case), but no condition by group interaction (F(1,92)⩽0.43, *P*⩾0.51 in each case). However, a main effect of condition was present in this contrast (F(1,92)⩾6.11, *P*⩽0.015, in each case), indicative of slower and more variable RT when multi-tasking for all children. This suggests that children with 16p11.2 deletion show differences in RT and RT variability relative to both their siblings and the unrelated neurotypical controls. As above, the primary effects of interest remain significant when Non-verbal IQ was used as an additional covariate in the ANCOVA analyses (F(1,33)⩾5.4, *P*⩽0.027).

### Non-adaptive traditional cognitive assessments: children with 16p11.2 deletion and neurotypical children

**Figure 3:**
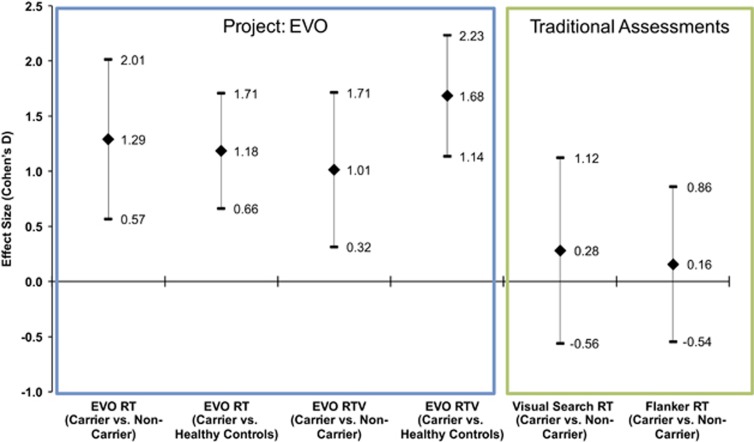
Illustration of effect sizes. Effect sizes (Cohen's D) for EVO and iPad assessments are displayed, with these values calculated from repeated measures estimated marginal means for group main effects. RT, response time.

We evaluated response time and response time variability performance for the Flanker and Visual Search selective attention tasks on all trial types and task measures between children with 16p11.2 deletion and unaffected children. Across all of these tests and measures, no significant group difference was observed (F⩽3.7, *P*⩾0.064, *d*⩽0.28) in each case; see Figures 2 and 3, Table 3 for depiction of these results). Given that there was a trend toward significance for the Flanker response time cost (incongruent RT−congruent RT), further examination of this result revealed that the carrier group actually exhibited an inverse cost (for example, they performed better on the harder condition of the Flanker task), a result that is inconsistent with design and utility of this task across a wealth of literature.^30^ The inclusion of Non-verbal IQ as an additional covariate in the ANCOVA analyses resulted in the same pattern of effects observed above.

## Discussion

The present findings demonstrated that children with 16p11.2 deletion show visuomotor and cognitive control deficits relative to age-matched non-carrier siblings and neurotypical unrelated children when using an adaptive, scientifically inspired digital platform for cognitive assessment. Furthermore, cognitive control deficits were not observed when using traditional non-adaptive assessments (Flanker and Visual Search). These results have two important implications: first, children with 16p11.2 deletion have selective attention deficits that likely affect their learning and real-world function, and second, these deficiencies may be overlooked if using traditional assessments.

The two traditional selective attention assessments used here have consistently shown differences between healthy and attentionally deficient groups,^45, 46, 47, 48, 49^ with similar effects seen when deployed on an iPad^42^ or internet browser^50^ (however, see Bauer *et al.*^51^ as a point of caution regarding assumptions of validity and reliability when digitally converting testing tools). However, these assessments failed to reveal group differences between the children with 16p11.2 deletion and their siblings. One may question whether the differential results between the adaptive versus non-adaptive assessments reflect variability in the cognitive challenge presented. Indeed, increased cognitive load has been shown to negatively affect performance in children,^52, 53^ young adults^54, 55, 56, 57^ and older adults.^56, 58, 59^ However, this does not appear to be the case here: no group differences emerged for the flanker task, nor any for the visual search task which has both a low and high selective attention load. These findings suggest that enjoyable technology that engages a participant with adaptive mechanics can reveal phenotypic differences in highly variable populations.

There is a general consensus that computerized response time measurements can act as a valuable indicator of cognitive ability.^60, 61, 62, 63^ Several studies have shown that response time variability is increased in children and older adults compared with younger adults.^61, 64, 65^ Furthermore, response time variability has been found to distinguish groups of individuals with and without ADHD,^66, 67, 68, 69^ as well as individuals on the autism spectrum.^38^ However, a recent meta-analysis by Karalunas *et al.*^38^ examining response time variability based on non-adaptive measures suggested these assessments only have small to moderate effect sizes (Hedges' *g*=0.37, interpreted similar to Cohen's *d*) when attempting to dissociate children with autism or ADHD from neurotypical controls. Here the observed between-group effect sizes for response time performance and variability using the EVO assessment (including the correction for sample size and covarying for age) were quite high (*d*⩾0.83). These findings suggest that the ability to detect group differences between populations that are inherently variable requires tools that demonstrate greater sensitivity. Even in cases for which sensitivity and specificity were found to be comparable between adaptive and non-adaptive platforms,^70, 71^ adaptive platforms have the added benefit of requiring less total testing time than ‘traditional' computerized testing, and are able to mitigate potential ceiling and floor effects,^17^ which is a concern for populations in which inter-individual variability is high.^72, 73, 74, 75^ Here the use of adaptive algorithms in concert with entertainment-based video game factors likely contributed to participants being truly engaged in the testing experience. Thus, the observed null between-group differences observed in some studies may be due to a lack of sensitivity in the measurement tools being used as a function of participant engagement.

Parallels can be made between the mechanisms underlying the response time/response time variability effects observed here and similar effects reported in distinct populations. The field of cognitive aging has associated increased response times and variability with neural dedifferentiation (for example, where both structure and function becomes less focal with age^76, 77^) which in turn leads to increased neural noise^78^ (a result also common to children with autism^79^). Recent neuroimaging findings involving children with the 16p11.2 deletion have demonstrated that these individuals have irregularities in their white matter tracts connecting brain regions^80^ that are indicative of improper differentiation. These findings are particularly intriguing given that deficient attentional resources have been associated with reduced frontal–posterior connectivity,^18^ with this measure also associated with increased response time variability in both children and older adults.^81^ These findings hint at the possibility that the impaired performance observed here stems from the children with the 16p11.2 deletion having less functional neural differentiation than their healthy counterparts at prefrontal regions, negatively affecting the generation of midline frontal theta activity for tasks requiring attentional resources. Future work examining these types of underlying neural correlates within this population would better elucidate the mechanisms associated with the impacted performance observed here.

Given the approach taken here, there are some clear limitations of this study. Of particular concern would be the sample size of the patient population is relatively small, the unrelated control group did not complete the non-adaptive assessments as they were involved in another research study, testing environments were inherently different between these groups, and we do not have a robust characterization of our sample that includes IQ due to the nature of how the data were collected (for example, at a family meeting). These factors must be taken into account when assessing the present findings, as those with higher IQs would be expected to have stronger selective attention abilities, and the observed group differences are subsumed in part by possible general cognitive delays. However, the carrier group is reasonably sized for a rare genetic event and genetically comparable since all the 16p11.2 deletion carriers have the same breakpoints and no other pathogenic copy-number variants. Furthermore, the differential effects observed between the adaptive and non-adaptive tests underscore the idea of using more sophisticated approaches to enhance testing sensitivity. One of the primary functions of the adaptive algorithms being incorporated is to ensure fair comparisons between groups with disparate abilities (in this case those with IQ differences). Although this approach would not remove the need and utility of collecting IQ data, it does provide the means for standardizing performance in situations where IQ would not be available. Another concern involves the identification of group differences in motor function or processing speed, or possible group differences in video game experience leading to the observed effects. While having these measures on each participant would be ideal, adaptivity facilitates having each participant in a personalized testing state during testing, allowing for participants to have differences in these abilities without contaminating subsequent between-group comparisons (for example, see Anguera *et al.*^18^ for a similar approach involving older adults).

Finally, one may question whether EVO is actually testing a distinct type of attention from that measured by the flanker and visual search tasks, or is inadvertently reflecting group differences associated with general familiarity with video games. The common thread across each task is the engagement of selective attention resources, with each task assessing this construct in a distinct manner. Although the EVO platform has a video game presence, the underlying task being performed is at its core a selective attention task, with the adaptivity mechanics directly ensuring that differences in video game experiences are mediated for subsequent group comparisons. Although there was no quantitative evaluation demonstrating that participants found EVO to be more engaging than the other assessments, it is critical to keep in mind that engagement is not the sole driving factor in creating a more sensitive testing environment. Indeed, a more enjoyable experience can lead to enhanced engagement through increased participant motivation; however, heightened testing sensitivity requires the proper incorporation of these factors in addition to the proper titration of adaptive mechanics.^1^ Furthermore, there may be situations where an individual with atypical attention would benefit from distinctly different testing environments that are less visually stimulating, providing further evidence that even the approach used here is not a panacea for all future assessment work. It should be noted that these adaptive mechanics are not directly related with making such an experience more enjoyable *per se*; however, ‘fun' can make a testing experience more sensitive by encouraging greater participant engagement. In summary, the present findings suggest that children with cognitive control impairments when optimally engaged reveal measurable deficits in response time and response time variability relative to neurotypical controls.

## Figures and Tables

**Table 1:** Task description

	*Project: EVO*	*Flanker task*	*Visual search task*
Task description		Indicate direction of central target, which is flanked by distractors that are either in the same or opposite direction as the target	Search for a target (green ‘Π'), among a number of red and non-vertically aligned green distracting ‘Π's, indicating the side of the Π opening being on the top or the bottom (as shown).
Primary Reference	Anguera *et al.*^18^	Eriksen and Eriksen^39^	Treisman (1982)
Type of Attention Measured	Selective attention with distractors, alone and while multi-tasking	Selective attention with distractors	Selective attention with distractors, with search component
Primary measure to assess attention	Response time to targets under differing distraction loads	Response time to congruent versus incongruent targets	Response time to targets under high versus low loads of distractors
Respond to select stimuli	Yes	Yes	Yes
High/Low Difficulty Loads	Yes	No	Yes
Distracting or Irrelevant Stimuli	Yes	Yes	Yes
Trial-by-Trial Feedback	Yes	Yes	Yes
Incorporates high-level art and music to create immersive experience	Yes	No	No
Uses Adaptivity	Yes	No	No
Involves multi-tasking	Yes	No	No

**Table 2:** Demographic profiles of participants^a^

	*Carriers* N=*13*	*Non-carrier siblings* N=*11*	*Unrelated controls* N=*24*	*F-value (*P*-value)*
*IQ (subset)*				
Verbal IQ	84.3±11.4	108.3±13.3	124.9±11.9	51.12 (<0.0001)
Non-verbal IQ	88.5±10.1	100.8±10.6	113.6±12	19.73 (<0.0001)
Full scale IQ	85.5±10.5	103.6±12.5	119.1±10.5	41.3 (<0.0001)
				
ADHD diagnosis	6 (46%)	2 (22%)	0	
Autism spectrum disorder	1 (8%)	1 (9%)	0	
Learning disorder	2 (15%)	0	0	
Anxiety disorder	0	1 (14%)	0	
Mood disorder	1 (8%)	0	0	
Age (full sample)	*N*=20	*N*=16	*N*=75	
	10.1±3	10.1±2.8	10.8±1.4	2.41 (0.054)
Gender (full sample)	6 female	7 female	34 female	1.54 (0.46)^b^

Abbreviations: ADHD, attention deficit/hyperactivity disorder; IQ, intellectual quotient.

a Cognitive assessments and clinical evaluations were conducted as part of the broader Simons Foundation 16p11.2 and UCSF Sensory Neurodevelopment and Autism study. Verbal IQ was calculated from the standard verbal reasoning score of the DAS and the verbal comprehension index of the WISC-IV. Non-verbal IQ is calculated from the standard non-verbal reasoning score of the DAS and the perceptual reasoning index of the WISC-IV. Full scale IQ is calculated from general conceptual ability score of the DAS and the full scale IQ composite score of the WISC-IV.

b Chi-square with 2 df.

**Table 3:** Between-group task descriptions

*Task*	*Trial type*	*Carrier*	*Non-carrier*^a^	
		*Group mean RT (s.d.)* N=*18*	*Group mean RT (s.d.)* N=*19*	
Visual search (ms)	Conjunction: 4-item	1175 (419)	1163 (400)	
	Conjunction: 12-item	1517 (863)	1616 (833)	
	Feature: 4-item	983 (327)	906.33 (315)	
	Feature: 12-item	971 (225)	926.4 (243)	
			
Flanker (ms)	Incongruent	657 (144)	754 (163)	
	Congruent	676 (125)	712 (140)	
			
		N=*20*	*Non-carrier unaffected siblings (*N=*16)*	*Non-related healthy controls (*N=*75)*
EVO (ms)	Single-tasking RT	603 (144)	487 (126)^b^	491 (80)^b^
	Multi-tasking RT	718 (178)	551 (95)^b^	591 (67)^b^

Abbreviation: RT, response time.

a 5 of these individuals where non-related healthy controls.

b *P*<0.05 between-group difference from the carrier group.
